# Accurate global potential energy surface for the ground state of CH_2_^+^ by extrapolation to the complete basis set limit†

**DOI:** 10.1039/c8ra02228c

**Published:** 2018-04-11

**Authors:** Lu Guo, Hongyu Ma, Lulu Zhang, Yuzhi Song, Yongqing Li

**Affiliations:** Department of Physics, Liaoning University Shenyang 110036 China yqli@lnu.edu.cn; School of Physics and Electronics, Shandong Normal University Jinan 250014 China yzsong@sdnu.edu.cn

## Abstract

A full three-dimensional global potential energy surface is reported for the ground state of CH_2_^+^ by fitting accurate multireference configuration interaction energies calculated using aug-cc-pVQZ and aug-cc-pV5Z basis sets with extrapolation of the electron correlation energy to the complete basis set limit. The topographical characteristics have been compared in detail with a potential energy surface of the same type recently reported [*J. Chem. Phys.*, 2015, **142**, 124302] based on a least-squares fit to accurate high level *ab initio* MRCI(Q) energies, calculated using AV6Z basis set. The new three-dimensional global potential energy surface is then used in quasiclassical trajectory calculations for H(^2^S) + CH^+^(*X*^1^Σ^+^) → C^+^(^2^P) + H_2_(*X*^1^Σ_g_^+^) reaction. The integral cross sections, differential cross sections and the rate coefficients have been computed. A comparison shows that our potential energy surface can be applied to any type of dynamic study.

## Abstract 2

A full three-dimensional global potential energy surface is reported for the ground state of CH_2_^+^ by fitting accurate multireference configuration interaction energies calculated using aug-cc-pVQZ and aug-cc-pV5Z basis sets with extrapolation of the electron correlation energy to the complete basis set limit.

## Graphical abstract

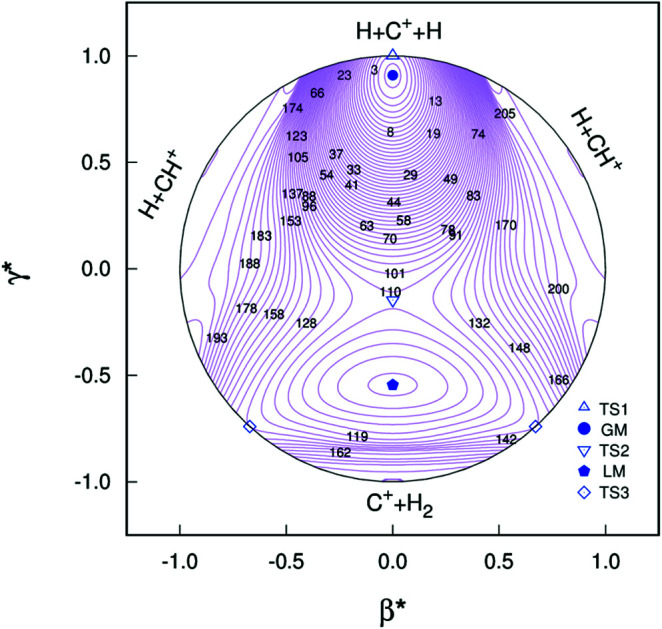

## 1. Introduction

The C^+^ + H_2_, ion-molecule reaction has been the research core of extensive experimental and theoretical study owing to its important role in astrophysics and atmospheric chemistry. Particularly, the CH_2_^+^ complex formed by the reaction is a crucial intermediate in interstellar matter and combustion process.^1^ Hence the reaction C^+^ + H_2_ has been widely researched.

The cation CH_2_^+^ belongs to a class of molecules which are termed ‘‘quasilinear’’. From the spectroscopic side, the linear and bending problems of CH_2_^+^ attracted the researchers' interest in the 1960s.^2^ This issue was first solved theoretically due to the lack of spectral data, Schaefer and Bender^3^ predicted a bent equilibrium geometry for the electronic ground state (1^2^A′) of CH_2_^+^ in 1971. Later, the bent equilibrium geometry was confirmed by the Coulomb explosion imaging experiment.^4,5^ Then, extensive research on CH_2_^+^ has been carried out theoretically.

Among a series of theoretical studies, Stoecklin and Halvick^6^ reported the theoretical research result of the caption reaction for the first time, which fitting a precise 3-D single-valued potential energy surface (PES). Utilizing an extensive multiconfigurational wave function with the augmented Dunning correlation consistent basis set (aug-cc-pVQZ) to calculate large *ab initio* points. Then Halvick *et al.*^7^ based on this PES to study quasiclassical trajectory (QCT) calculations, along with phase space theory and quantum rigid rotor calculations for H + CH^+^. And the PES was used by Zanchet *et al.*^8^ for the state-to-state rate constant study with quantum wave packet analysis of the H(^2^S) + CH^+^(*X*^1^Σ^+^) → C^+^(^2^P) + H_2_(*X*^1^Σ_g_^+^) reaction. Recently, Schneider and Warmbier^9^ constructed the CH_2_^+^ ground state PES by fitting internuclear distances polynomials with the multireference configuration interaction (MRCI) and aug-cc-pVTZ *ab initio* points. And for verifying this PES, they have performed quantum scattering and QCT calculations. Additionally, Herráez–Aguilar *et al.*^10^ have performed a dynamical study of the endothermic and barrierless C^+^ + H_2_(^1^Σ_g_^+^) → CH^+^(^1^Σ_g_^+^) + H reaction for different initial rotational states of the H_2_(*v* = 0) and H_2_(*v* = 1) manifolds, with the QCT and the Gaussian binning methodology on Schneider and Warmbier's^9^ PES. Most recently, Li *et al.*^11^ reported a new many-body expansion (MBE) PES by fitting MRCI/aug-cc-pV6Z *ab initio* energies. This PES is used by Guo *et al.*^12^ to analyze the effect of isotopic substitution on three-dimensional dynamic properties of the reactions C^+^ + H_2_/HD/HT → CH^+^ + H/D/T. In addition, the time-dependent wave packet propagation approach was used to compute thermal rate constants and integral cross sections of the H + CH^+^ reaction in the coupled states approximation by Sundaram *et al.*^13^ Faure *et al.* also presents a detailed theoretical study of state-to-state and initial-state-specific rate coefficients are computed in the kinetic temperature range 10–3000 K.^14^

The purpose of present study is to build a high quality global PES for the ground state(1^2^A′) of CH_2_^+^ from MRCI(Q)^15^*ab initio* energies based on the reference full valence complete active space (FVCAS)^16^ wave function, the aug-cc-pV5Z(AV5Z) and aug-cc-pVQZ(AVQZ) basis sets of Dunning^17,18^ have been applied. We all know that in order to get a highly precise PES, it usually requires a large basis sets. But, we did not use such a large basis sets in this work, instead we have extrapolated the total energy to the complete basis set (CBS) limit by using a uniform single-pair and triple-pair (USTE).^19,20^ For verifying this PES, the QCT has been performed on H(^2^S) + CH^+^(*X*^1^Σ^+^) → C^+^(^2^P) + H_2_(*X*^1^Σ_g_^+^), the differential cross sections (DCSs), integral cross sections (ICSs) and the rate coefficients will be computed.

The paper is organized as follows. Section 2 introduces the theoretical approaches, such as *ab initio* calculations and the application of extrapolation. Section 3 introduces the analytic expression of CH_2_^+^(1^2^A′) PES. Main topographical features are discussed in section 4. Section 5 describes the QCT calculations. Finally, the conclusion is presented in section 6.

## 2. *Ab initio* calculations and extrapolation scheme

The MRCI(Q)^15^ approach is one of the best methods to obtain the precise PESs. All *ab initio* calculations are performed at the MRCI(Q) level with the FVCAS^16^ as reference. MOLPRO 2012 (ref. 21) is a kind of program package about the quantum chemistry, in association with the Dunning *et al.*^17,18^ correlation-consistent basis sets have been applied during our work. This procedure involves 6 active orbitals (5A′ + 1A′′), with a total of 443 (166A′ + 177A′′) configuration state functions at AV5Z and AVQZ basis sets, respectively. In all 3255 *ab initio* grid points have been computed for C^+^–H_2_ channels, the region was defined by 1.2 ≤ *R*_H_2__/*a*_0_ ≤ 4.4, 1.4 ≤ *r*_C^+^–H_2__/*a*_0_ ≤ 10 and 0 ≤ *γ*/deg ≤ 90, while, for H–CH^+^, they cover geometries defined by 1.8 ≤ *R*_CH^+^_/*a*_0_ ≤ 3.6, 1.4 ≤ *r*_H–CH^+^_/*a*_0_ ≤ 10 and 0 ≤ *γ*/deg ≤ 180, *R*, *r* and *γ* are atom-diatom Jacobi coordinates for both channels. To get more precise energy points, the USTE^19,20^ method is adopted. During the calculations, the core is frozen and ignoring the relativistic effect.

In order to carry out the extrapolation, electronic energy in the MRCI(Q) calculation is expressed by a sum of two terms^19^

1 *E*_x_ = *E*^CAS^_x_ + *E*^dc^_x_,

where the superscript CAS represents the complete-active space and the superscript dc represents the dynamical correlation energies, in addition the subscript X signifies that the electronic energy computed in the AVXZ basis set. The X = Q, 5 are used during the calculation.

Using a two-point extrapolation program suggested by Karton and Martin(KM),^22^ the CAS energies are extrapolated to the CBS limit

2 *E*^CAS^_x_ = *E*^CAS^_∞_ + *B*/*X*^α^,

where *E*^CAS^_x_ is the energy when *X* → ∞ and *α* = 5.34 is an effective decay index.

The USTE protocol^19,20^ has been triumphantly implemented to extrapolate the dynamical correlation energies in MRCI(Q) calculations, which is extrapolated by the formula3
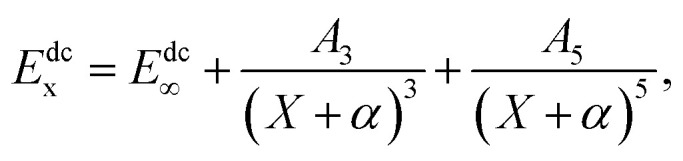
where *A*_5_ is written as the auxiliary relation

4 *A*_5_ = *A*_5_(0) + *cA*_3_^5/4^

With *α* = −3/8, *c* = −1.17847713 and *A*_5_(0) = 0.0037685459 are “universal-like” parameters.^19^Eqn (3) could be converted to (*E*_∞_, *A*_3_) two-parameter rule, which has access to the actual extrapolation process.

## 3. Analytical potential energy function of CH_2_^+^(1^2^A′)

The analytical function of CH_2_^+^(1^2^A′) PES can be represented as a MBE^23,24^ form5
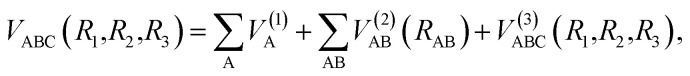
where *V*_A_^(1)^ is the isolated atomic energy, *V*_AB_^(2)^ is a two-body energy term and *V*_ABC_^(3)^ is the three-body energy term which is zero at all dissociation limits. In this work, the title system obeys the following dissociation scheme:6
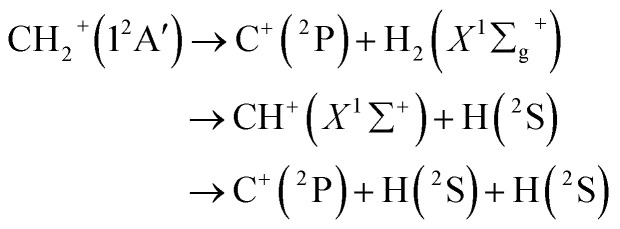
where C^+^(^2^P) and H(^2^S) are all in their ground states. So, the one-body energy term *V*_A_^(1)^ in eqn (5) are zero.

### A. Two-body energy terms

The analytic energy function of the two-body terms *V*_AB_^(2)^ for CH^+^(*X*^1^Σ^+^) and H_2_(*X*^1^Σ_g_^+^) are imitated employing the Aguado and Paniagua^25,26^ approach, which the function for title diatomic systems can be represented as summation of the short-range and long-range potentials

7 *V*^(2)^_AB_ = *V*^(2)^_short_ + *V*^(2)^_long_

where8
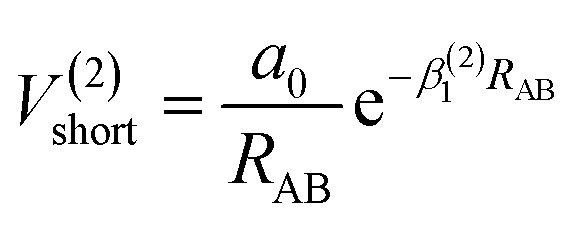
which the potential energy of diatomic tends to infinitely great when *R*_AB_ → 0. The long-range potential is expressed as9
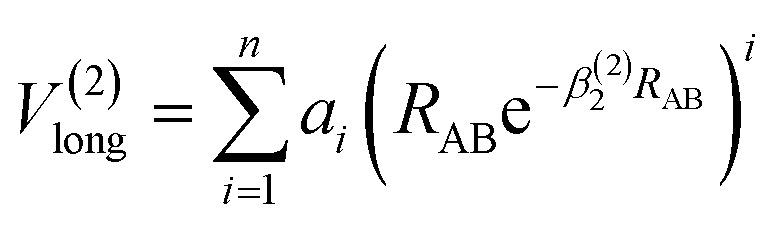
which the potential energy of diatom is equal to zero as *R*_AB_ → ∞. The potential function in eqn (9) is truncated up to 8th power (*n* = 8), we can obtain 11 parameters, including 2 nonlinear parameters *β*_*i*_(*i* = 1, 2) and 9 linear parameters *a*_*i*_(*i* = 0, 1,…, 8) for CH^+^(*X*^1^Σ^+^) and H_2_(*X*^1^Σ_g_^+^) by the fitting procedure. All the fitted parameters of the CH^+^(*X*^1^Σ^+^) and H_2_(*X*^1^Σ_g_^+^) are listed in Table 1 of the ESI.†

### B. Three-body energy term

For the calculation of three-body term, the method of three-body distributed polynomial is adopted,^27,28^ which was applied to calculate ground and excited states of FH_2_,^29^ NH_2_ (ref. 30–32) and NH_3_.^33,34^10
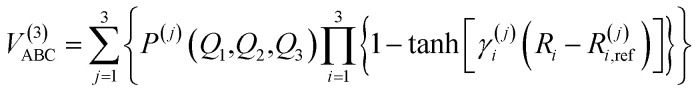
where *P*^(*j*)^(*Q*_1_,*Q*_2_,*Q*_3_) is the *j*-th (*j* = 1, 2, 3) polynomial, with *Q*_*i*_ (*i* = 1, 2, 3) being the symmetric coordinates, *γ*_*i*_^(*j*)^ are determining parameters of the nonlinear range and *R*_*i*,ref_^(*j*)^ represent reference geometries. So, there are 150 linear coefficients, 9 reference bond distances and 9 nonlinear ones in all. In this work, a total of 3255 CBS points has been calculated in fitting procedure and fitting result shows that the total root mean square derivation is rmsd = 0.55 kcal mol^−1^. All the fitted parameters of the least square method are listed in Tables 2 and 3 of the ESI.†

## 4. Features of the PES

**Fig. 1:**
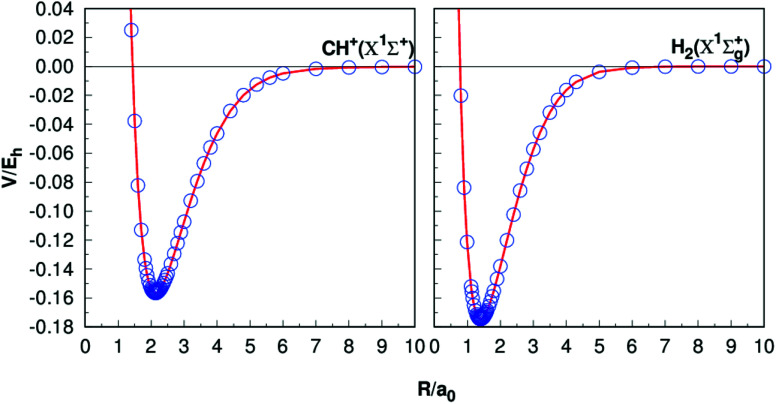
Potential energy curves of CH^+^(*X*^1^Σ^+^) and H_2_(*X*^1^Σ_g_^+^). The circles indicate the CBS(Q,5) energies.

Table 1 presents the results of *R*_e_, *D*_e_, *ω*_e_, *ω*_e_*χ*_e_, *α*_e_ and *B*_e_ of CH^+^(*X*^1^Σ^+^) and H_2_(*X*^1^Σ_g_^+^) together with the other theoretical^11,35–37,40–42^ and experimental^38–42^ data. The equilibrium internuclear *R*_e_ of CH^+^(*X*^1^Σ^+^) to be 2.135*a*_0_, which is only 0.002*a*_0_ shorter than the experimental result^38^ and 0.001*a*_0_ shorter than the latest result computed by Li *et al.*,^11^ while for the case of H_2_, all the results are 1.401*a*_0_,^40,42−44^ it shows a high precision. For the dissociation energies *D*_e_, CH^+^(*X*^1^Σ^+^) differs from the theoretical and experimental results by less than 0.005 eV.^11,39^ For the H_2_ (*X*^1^Σ_g_^+^) has dissociation energy *D*_e_ = 4.749 eV, this compares well with the corresponding theoretical values are *D*_e_ = 4.748 eV (ref. 11) and corresponding experimental values *D*_e_ = 4.478 eV.^45^ Overall, it can be concluded that the other spectroscopic constants are in good agreement with these literature results. Fig. 1 shows the potential energy curves (PECs) of CH^+^(*X*^1^Σ^+^) and H_2_(*X*^1^Σ_g_^+^). In order to evaluate the quality of the fitting, we calculate the rmsd. The rmsd of CH^+^(*X*^1^Σ^+^) and H_2_(*X*^1^Σ_g_^+^) PECs are 0.05 kcal mol^−1^ and 0.005 kcal mol^−1^, respectively. As a whole, it reveals a high quality fitting process. Shown in Fig. 1, the PECs at the CBS/USTE(Q,5) calculations nicely, showing accurate and smooth behavior both in short and long regions.

**Table 1:** Spectroscopic constants of CH^+^ and H_2_ diatoms, with the unit of *R*_e_ in *a*_0_, *D*_e_ in eV and *ω*_e_, *ω*_e_*χ*_e_, *α*_e_ and *β*_e_ in cm^−1^

	*R*_e_	*D*_e_	*ω*_e_	*ω*_e_ *χ*_e_	*α*_e_	*β*_e_
**CH** ^**+**^ **(*X*** ^**1**^ **Σ** ^**+**^ **)**						
This work	2.135	4.257	2861.948	59.629	0.447	14.311
Theor.^11^	2.136	4.252	2853.027	58.515	0.489	14.201
Theor.^35^	2.136	4.244	2851	58.1	0.489	14.199
Theor.^36^	2.144	—	2849.03	66.448	0.490	14.094
Theor.^37^	2.127	4.660	3111.0	38.44	—	—
Exp.^38^	2.137	4.26 ^39^	2857.56	59.32	0.495	14.178

**H**_**2**_ **(*X*** ^**1**^ **Σ**_**g**_^**+**^ **)**						
This work	1.401	4.749	4404.613	126.636	2.233	60.861
Theor.^40^	1.401	4.748	4403.60	126.602	2.232	60.864
Theor.^41^	1.403	4.748	4395.22	126.119	2.221	60.735
Theor.^42^	1.401	4.711	4389.66	121.560	3.162	60.826
Exp.^43^	1.401	4.478 ^45^	4401.21	121.33	3.062	60.853
Exp.^44^	1.401	4.476	4395.20	117.99	2.993	60.809

Table 2 collects all known stationary points (geometry, energy and vibrational frequencies) of the new PES for ground state of CH_2_^+^. For better comparisons, the results of other theoretical and experimental work reports are also gathered in Table 2. A global minimum (GM) is found to local at *θ*_HC^+^H_ = 138.6°, *R*_1_ = 3.859*a*_0_ and *R*_2_ = *R*_3_ = 2.063*a*_0_ with the minimum energy −0.333*E*_h_ relative to the all dissociation asymptote (*R*_1_ represents the H_2_ interatomic separation, while *R*_2_ and *R*_3_ represent the two CH^+^ interatomic separations), its maximum deviation is only 0.004*a*_0_ for the CH^+^ bond length (*R*_2_ and *R*_3_) when compared to the data of the MRCI(Q)/AV6Z PES^11^ with the *θ*_HC^+^H_ = 141.0°, *R*_1_ = 3.896*a*_0_ and *R*_2_ = *R*_3_ = 2.067*a*_0_. Comparing with the theoretical works of Stoecklin and Halvick,^6^*R*_1_ = 3.865*a*_0_ is 0.006*a*_0_ longer than our results. This compares well with the corresponding experimental values^2,46^ of *θ*_HC^+^H_ = 139.8°. For the harmonic frequencies, the PES from this work computes values of 3048 cm^−1^, 3272 cm^−1^ and 921 cm^−1^, there is good consistency with the theoretical results calculated by Brinkmann *et al.*^47^, which are 3011 cm^−1^, 3260 cm^−1^ and 965 cm^−1^, respectively. Table 2 also collects the attributes of a local minimum (LM) and three transition states: TS1(*D*_∞h_), TS2(*C*_2v_) and TS3(*C*_∞v_) barriers.

**Table 2:** Stationary points at the valence region for ground state of CH_2_^+^ PES, with the unit of *R*_1_, *R*_2_ and *R*_3_ in *a*_0_, *θ*_HCH_ in deg, Δ*V* in kcal mol^−1^, *ω*_1_, *ω*_2_ and *ω*_3_ in cm^−1^

Feature	*R*_1_	*R*_2_	*R*_3_	*θ*_HCH_	Δ*V*^a^	*ω*_1_	*ω*_2_	*ω*_3_
**Global minimum**								
This work	3.859	2.063	2.063	138.6	−99.64	3048	3272	921
Theor.^11^	3.896	2.067	2.067	141.0	−99.54	2983	3283	957
Theor.^47^	3.890	2.067	2.067	140.39	—	3011	3260	965
Theor.^6^	3.865	2.075	2.075	137.3	—	—	—	—
Exp.^2,46^	3.922	2.088	2.088	139.8	—	—	3133.4	—

**Local minimum**								
LM(*C*_2v_)	1.648	2.757	2.757	34.8	−29.63	3254	1317	1220
Theor.^11^	1.666	2.645	2.645	36.7	−29.28	2937	1444	1307

**Transition state**								
TS1(*D*_∞h_)	4.099	2.050	2.050	180.0	−97.13	3083	5489	1077i
Theor.^11^	4.127	2.064	2.064	180.0	−96.54	2939	5431	1319i
TS2(*C*_2v_)	2.106	2.362	2.362	52.9	−21.44	2265i	1766	1493
Theor.^11^	2.170	2.435	2.435	52.9	−23.41	2149i	2179	1745
TS3(C_∞v_)	1.525	2.660	4.185	0.0	−15.96	3557	664i	1527
Theor.^11^	1.511	2.645	4.156	0.0	−16.89	3676	172i	1769

a Relative to the C^+^ + H_2_ asymptote.

**Fig. 2:**
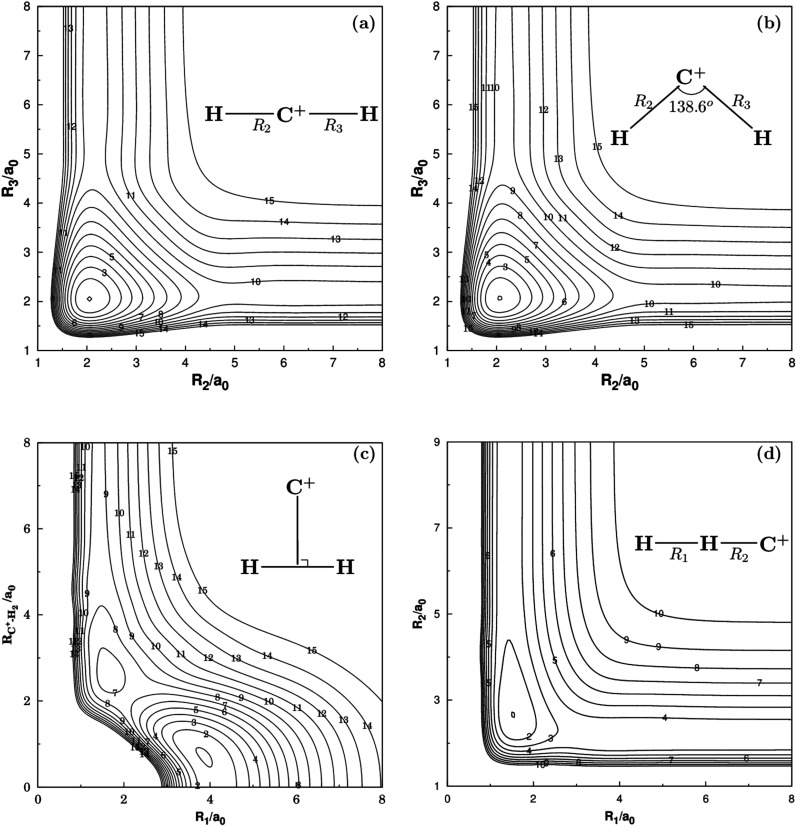
(a) Contour plot for bond stretching for linear [H–C–H]^+^ configurations. (b) Contour plot for bond stretching in [H–C–H]^+^, keeping the included angle fixed at 138.6°. (c) Contour plot for the *C*_2v_ insertion of the C^+^ into H_2_. (d) Contour plot for bond stretching in linear [H–H–C]^+^ configurations. Contours are equally spaced by 0.02*E*_h_, starting at −0.329*E*_h_ for panels (a), −0.333*E*_h_ for panels (b), −0.332*E*_h_ for panels (c) and −0.199*E*_h_ for panels (d).

**Fig. 3:**
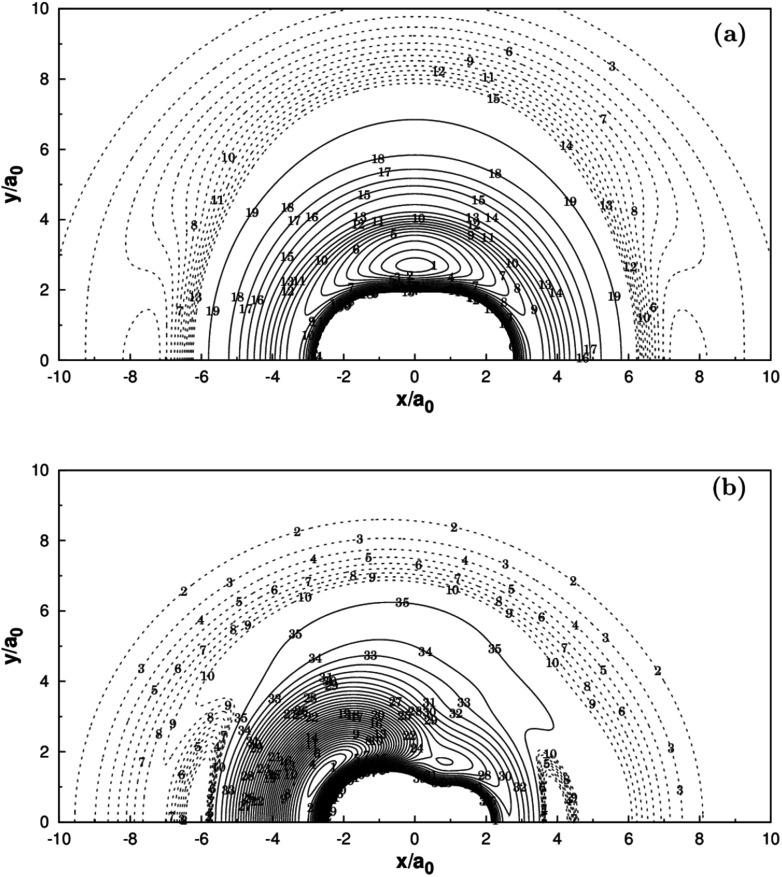
(a) Contour plot for a C^+^ moving around a H_2_ molecule fixed at the equilibrium geometry *R*_H_2__ = 1.401*a*_0_ and lying along the *X*-axis with the center of the bond fixed at the origin. Contours are equally spaced by 0.002*E*_h_, starting at −0.213*E*_h_. Shown in dash are contours equally spaced by −0.0001*E*_h_, starting at −0.174508*E*_h_. (b) Contour plot for a H atom moving around a CH^+^ fixed at the equilibrium geometry *R*_CH^+^_ = 2.135*a*_0_ and lying along the *X*-axis with the center of the bond fixed at the origin. Contours are equally spaced by 0.005*E*_h_, starting at −0.33*E*_h_. Shown in dash are contours equally spaced by −0.0002*E*_h_, starting at −0.156468*E*_h_.

Fig. 2 and 3 show the main topographical characteristics of the new CH_2_^+^ PES computed in this work. Obviously, there is a correct and smooth behavior in the entire configuration space. The salient features of these contour maps corresponding to several important stationary points for the main reaction. Fig. 2(a) illustrates a contour map for linear [H–C–H]^+^ stretch. The significant characteristic of this map is that there is a TS1(*D*_∞h_) linear transition state at *R*_2_ = *R*_3_ = 2.050*a*_0_ with an energy of 878 cm^−1^ above the GM of CH_2_^+^ but still 33 972 cm^−1^ below the energy of the C^+^ + H_2_ asymptote. This compares well with the MRCI(Q)/AV6Z PES,^11^ where the transition state is computed to local at *R*_2_ = *R*_3_ = 2.064*a*_0_ with an energy of 1050 cm^−1^ above the GM and 33 765 cm^−1^ below the reactants asymptote. The corresponding infrared spectrum^48^ result for this linear barrier is 1089 cm^−1^.

Fig. 2(b) plots for the bond stretching in [H–C–H]^+^ which the angle is fixed at138.6°. It can be found from Fig. 2(b) that there is a deep well for CH_2_^+^ PES, which is GM. Fig. 2(c) shows the contour plots to the insertion of C^+^ + H_2_ reaction. In this figure, the stationary points which are corresponding to TS1(*D*_∞h_), TS2(*C*_2v_), the LM and the GM. As shown in Table 2, the LM is predicted to locate at *R*_1_ = 1.648*a*_0_ and *R*_2_ = *R*_3_ = 2.757*a*_0_, so agreeing with MRCI(Q)/AV6Z PES.^11^ The main characteristics of the new PES for collinear [H–H–C]^+^ stretch are shown in the contour map of Fig. 2(d). The collinear TS3(*C*_∞v_) is found to locate at *R*_1_ = 1.525*a*_0_ and *R*_2_ = 2.660*a*_0_ with the energy of 15.96 kcal mol^−1^. This compares well with the *R*_1_ = 1.511*a*_0_ and *R*_2_ = 2.645*a*_0_ and 16.89 kcal mol^−1^ for the MRCI(Q)/AV6Z PES.^11^

Panel (a) of Fig. 3 illustrates the contour plot of C^+^ atom moving around H_2_(*X*^1^Σ_g_^+^) which the bond length at equilibrium geometry *R*_H_2__ = 1.401*a*_0_. Diatoms follow the *X*-axis centered on the origin. In addition, the corresponding contour plot of H atom moving around a fixed CH^+^(*X*^1^Σ^+^) is shown in (b) of the same figure, which the bond length is fixed at equilibrium geometry *R*_CH^+^_ = 2.135*a*_0_, which is in very good agreement with the MRCI(Q)/AV6Z PES.^11^ The two plots clearly show that there is a smooth behavior both in the long and short range.

All the main topographical characteristics of CH_2_^+^ PES can be also viewed in a relaxed triangular plot^49^ using scaled hyper-spherical coordinates (γ* = *γ*/*Q* and β* = *β*/*Q*), where the *Q*, *γ* and *β* are written as11
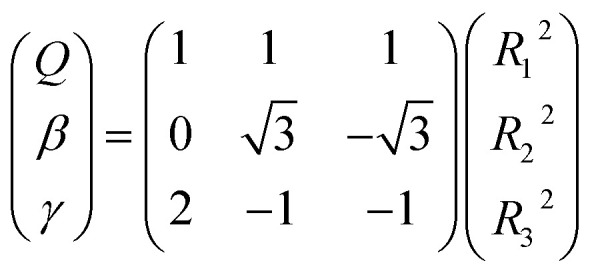


**Fig. 4:**
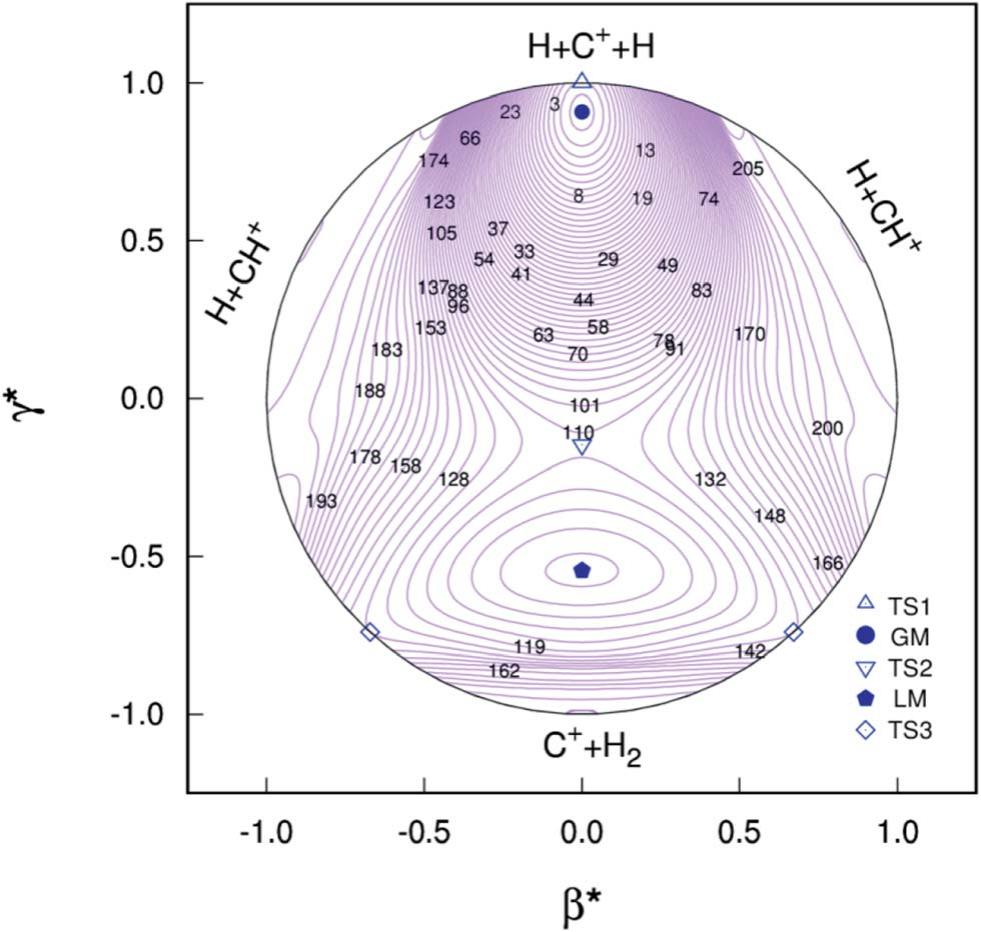
Relaxed triangular plot of the new PES of the present work in hyperspherical coordinates. Contours are equally spaced by 0.003*E*_h_, starting at −0.332*E*_h_. Also indicated are the various stationary points.

Clearly visible in Fig. 4 are all stationary points discussed above, which correspond to a GM, a LM and three transition states: TS1(*D*_∞h_), TS2(*C*_2v_) and TS3(*C*_∞v_) barriers.

**Fig. 5:**
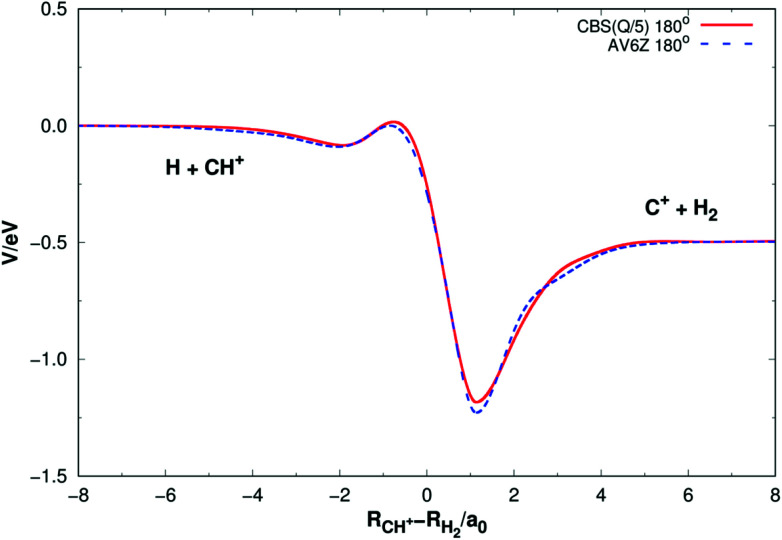
Minimum energy paths for H(^2^S) + CH^+^(*X*^1^Σ^+^) → C^+^(^2^P) + H_2_(*X*^1^Σ_g_^+^) reaction obtained from both the new PES and MRCI(Q)/AV6Z PES^11^ as a function of *R*_CH^+^_–*R*_H_2__. Collinear configuration ∠HC^+^H = 180°.

Fig. 5 shows the minimum energy paths (MEPs) for H(^2^S) + CH^+^(*X*^1^Σ^+^) → C^+^(^2^P) + H_2_(*X*^1^Σ_g_^+^) reaction obtained from both the new PES and MRCI(Q)/AV6Z PES^11^ for the collinear configuration ∠HC^+^H = 180°. The MEPs indicate the potential energy of CH_2_^+^ as a function for corresponding reaction coordinate of *R*_CH^+^_–*R*_H_2__, *R*_CH^+^_ and *R*_H_2__ as the internuclear distance between C^+^–H and H–H, respectively. As shown Fig. 5, there is a little barrier connecting a deep well and a shallow well. For the new PES, the relatively deeper well is found to locate at *R*_CH^+^_ = 2.660*a*_0_ and *R*_H_2__ = 1.525*a*_0_ and the little barrier locates at *R*_CH^+^_ = 2.210*a*_0_ and *R*_H_2__ = 2.972*a*_0_. The well depth and the barrier height are computed to be 1.184 eV and 0.016 eV. Comparing with the MRCI(Q)/AV6Z PES,^11^ the relatively deeper well locates at *R*_CH^+^_ = 2.645*a*_0_ and *R*_H_2__ = 1.511*a*_0_ and the little barrier is found to locate at *R*_CH^+^_ = 2.196*a*_0_ and *R*_H_2__ = 3.044*a*_0_. The well depth and the barrier height are computed to be 1.223 eV and 0.006 eV. Moreover, the reaction H(^2^S) + CH^+^(*X*^1^Σ^+^) → C^+^(^2^P) + H_2_(*X*^1^Σ_g_^+^) is exoergic by 0.49 eV base on the both PESs. It can be seen from Fig. 5, the results of the two PESs are in good agreement.

## 5. Dynamics of H + CH^+^ reaction

On the new PES, QCT^50,51^ calculation was performed for the H(^2^S) + CH^+^(*X*^1^Σ^+^) → C^+^(^2^P) + H_2_(*X*^1^Σ_g_^+^) reaction. In this work, we computed the ICSs, DCSs and rate constants. A total of 10 000 trajectories have been run for each of the collision energy. The time integration step is chosen to be 0.1 fs of classical motion equations, with H atom and the center of mass of the CH^+^ initially separated by 15.0 Å. The ICS is then written as12
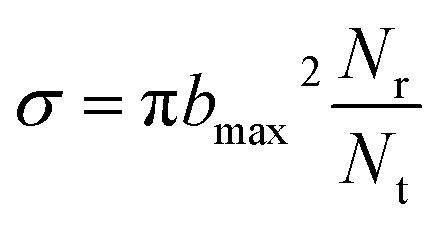
where *b*_max_ is the maximum impact parameter, *N*_r_ is the number of trajectories that go into a certain reaction channel and *N*_t_ is the total number of trajectories.

**Fig. 6:**
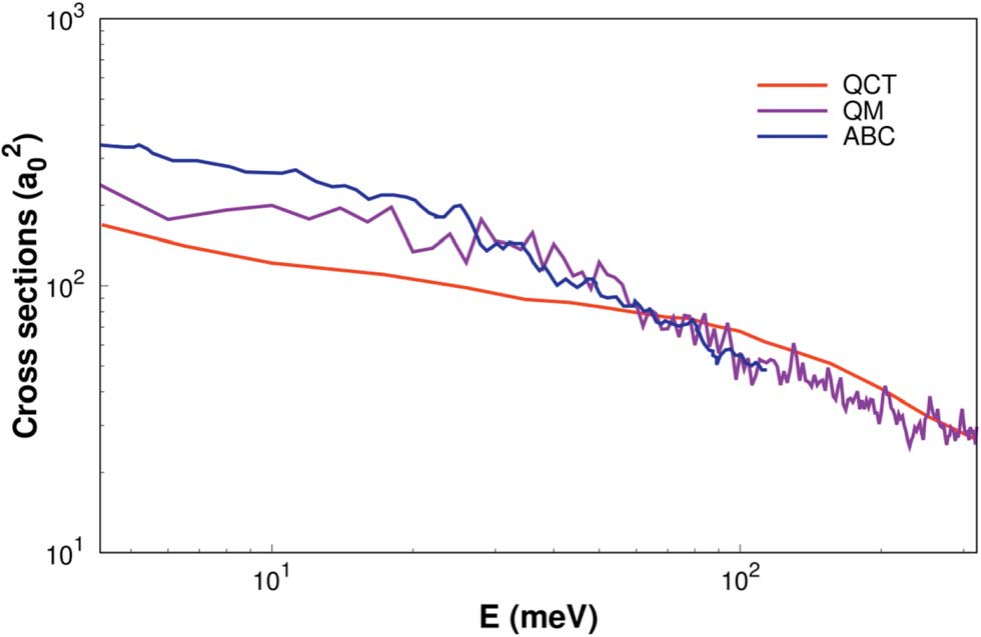
Integral cross sections of reaction of H(^2^S) + CH^+^(*X*^1^Σ^+^) → C^+^(^2^P)+ H_2_(*X*^1^Σ_g_^+^) as a function of collision energy. The QM and ABC from Li^11^ and Schneider^9^ are shown as well.

As shown Fig. 6, the ICSs are expressed as a collision energy function. For comparison our results with the quantum wave packet calculations^11^ and a modified version of the ABC^52^ quantum scattering code method have also been performed for the same reaction. We can find that the ICS based on our surface smaller than the results based on MRCI(Q)/AV6Z PES^11^ when the collision energy is less than about 40 meV. But when the collision energy is larger than about 40 meV, our results are consistency with quantum wave packet results. Overall, our results are reasonably good consistency with previous results.^11,52^

**Fig. 7:**
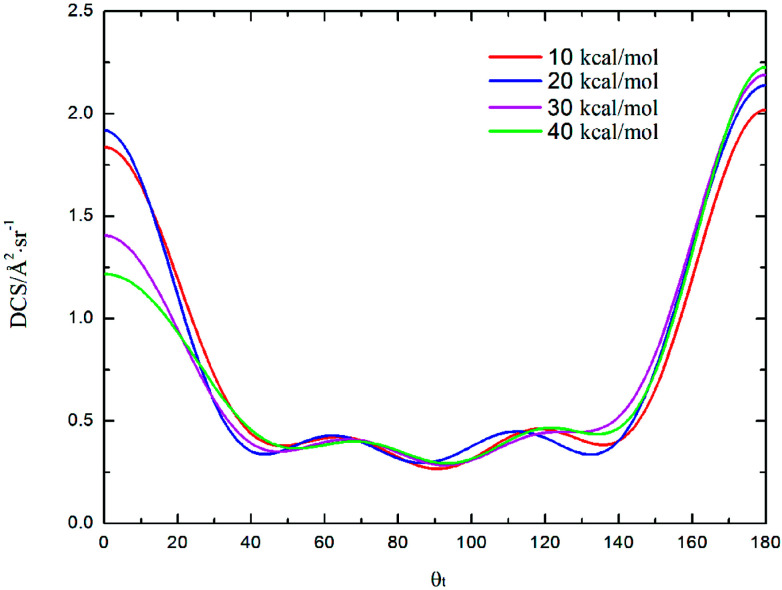
Differential cross section as a function of the scattering angle *θ*_t_ for the title reaction at the collision energy of 10, 20, 30 and 40 kcal mol^−1^.

DCS is mainly used to study product and reagent relative velocity ***k*–*k*′**, which is the most common vector correlation. The global angular distributions of H(^2^S) + CH^+^(*X*^1^Σ^+^) → C^+^(^2^P) + H_2_(*X*^1^Σ_g_^+^) reaction at collision energies of 10, 20, 30 and 40 kcal mol^−1^ based on the new PES are shown in Fig. 7. We can find that with the collision energies increase, the backward scattering phenomenon becomes more and more obvious. The enhanced phenomenon of backward scattering may be caused by an insertion reaction mechanism proposed by Pino *et al.*^53^

**Fig. 8:**
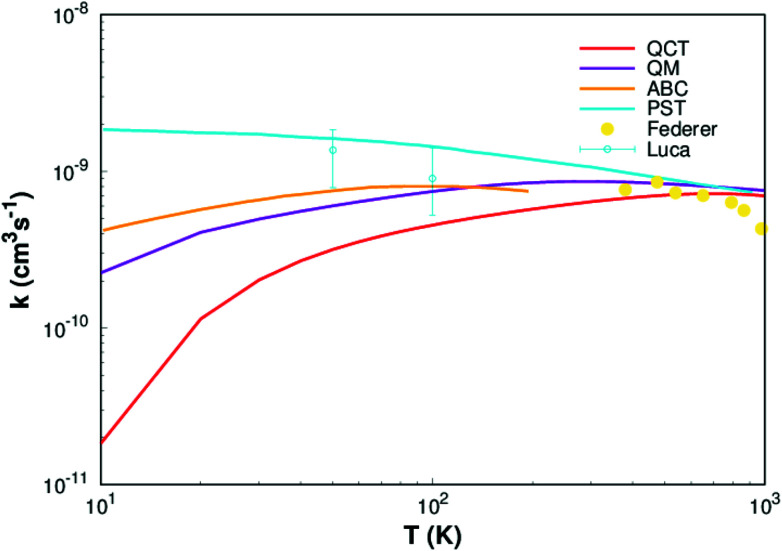
Rate constant for H(^2^S)+ CH^+^(*X*^1^Σ^+^) → C^+^(^2^P)+ H_2_(*X*^1^Σ_g_^+^) reaction calculated in this work. Other theoretical works of Li,^11^ Warmbier and Schneider^9^ and Halvick *et al.*,^7^ as well as experimental data from Luca *et al.*^55^ and Federer *et al.*^56^ are also included.

Finally, rate constants for the reaction H(^2^S) + CH^+^(*X*^1^Σ^+^) → C^+^(^2^P) + H_2_(*X*^1^Σ_g_^+^) are computed over the temperature range 10–1000 K by running QCT on the new PES of this work. By supposing a Maxwell–Boltzmann distribution on the collision energies, the rate constant is written as^54^13
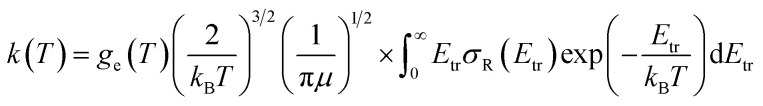
where *g*_e_ is the electronic degeneracy factor, we adopt *g*_e_ = 1 in the present work. By comparing with the results of various theoretical^7,9,11^ and experimental^55,56^ studies, the results are shown in the Fig. 8. It can be seen from Fig. 8 that our result (QCT) is less than others work for the whole temperature range. The QCT calculation is defect, the error will be larger when the collision energy is low, so the error of rate constant is also very large when the temperature is very low. However, as the temperature increases, the images get closer and closer to the other results, especially at high temperatures it agrees well with Li *et al.*^11^ and Federer *et al.*^56^ So, it turns out that our new PES can be applied to any type of dynamic study.

## 6. Conclusions

In our research process, we have constructed a high quality global PES for the ground state of CH_2_^+^(1^2^A′) from MRCI *ab initio* energies based on the reference FVCAS wave function, both AVQZ and AV5Z basis sets subsequently extrapolated to the CBS limit. All known stationary points including geometries, energies and vibrational frequencies can be obtained, and all the results are consistency with the corresponding theoretical and experimental values. The consistency and accuracy of the CBS method have also been affirmed by comparing the MRCI(Q)/AV6Z PES.^11^ Finally, QCT calculation has been performed on H(^2^S) + CH^+^(*X*^1^Σ^+^) → C^+^(^2^P) + H_2_(*X*^1^Σ_g_^+^), the ICS, DCS and the rate coefficients are computed in detail and compared to the MRCI(Q)/AV6Z and other PESs, as well as experimental values in the literature. In summary, the new PES built here can be used for any type of dynamic study.

## Conflicts of interest

There are no conflicts to declare.

## Supplementary Material

RA-008-C8RA02228C-s001

## References

[cit1] Herbst E. (1995). Annu. Rev. Phys. Chem..

[cit2] Willitsch S., Merkt F. (2003). J. Chem. Phys..

[cit3] Bender C. F., Schaefer H. F. (1971). J. Mol. Spectrosc..

[cit4] Graber T., Kanter E. P., Vager Z., Zajfman D. (1993). J. Chem. Phys..

[cit5] Baer A., Grieser M., Knoll L., Levin J., Repnow R., Schwalm D., Vager Z., Wester R., Wolf A., Zajfman D. (1999). Phys. Rev. A.

[cit6] Stoecklin T., Halvick P. (2005). Phys. Chem. Chem. Phys..

[cit7] Halvick P., Stoecklin T., Larrgaray P., Bonnet L. (2007). Phys. Chem. Chem. Phys..

[cit8] Zanchet A., Godard B., Bulut N., Roncero O., Halvick P., Cernicharo J. (2013). Astrophys. J..

[cit9] Warmbier R., Schneider R. (2011). Phys. Chem. Chem. Phys..

[cit10] Herráez-Aguilar D., Jambrina P. G., Menéndez M., Aldegunde J., Warmbier R., Aoiz F. J. (2014). Phys. Chem. Chem. Phys..

[cit11] Li Y. Q., Zhang P. Y., Han K. L. (2015). J. Chem. Phys..

[cit12] Guo L., Yang Y. F., Fan X. X., Ma F. C., Li Y. Q. (2017). Commun. Theor. Phys..

[cit13] Sundaram P., Manivannan V., Padmanaban R. (2017). Phys. Chem. Chem. Phys..

[cit14] Faure A., Halvick P., Stoecklin T., Honvault P., Epée Epée M. D., Mezei J. Z., Motapon O., Schneider I. F., Tennyson J., Roncero O., Bulut N., Zanchet A. (2017). MNRAS.

[cit15] Werner H. J., Knowles P. J. (1988). J. Chem. Phys..

[cit16] Knowles P. J., Werner H. J. (1985). Chem. Phys. Lett..

[cit17] Dunning Jr T. H. (1989). J. Chem. Phys..

[cit18] Woon D., Dunning T. H. (1993). J. Chem. Phys..

[cit19] Varandas A. J. C. (2007). J. Chem. Phys..

[cit20] Varandas A. J. C. (2007). J. Chem. Phys..

[cit21] Werner H. J. , Knowles P. J., Knizia G., Manby F. R. and Schutz M.and others, MOLPRO Version 2012.1, 2012, see http://www.molpro.net

[cit22] Karton A., Martin J. M. L. (2006). Theor. Chem. Acc..

[cit23] Murrell J. N. , Carter S., Farantos S. C., Huxley P. and Varandas A. J. C., Molecular potential energy functions, Wiley, Chichester, 1984

[cit24] Varandas A. J. C. (1988). Adv. Chem. Phys..

[cit25] Aguado A., Paniagua M. (1992). J. Chem. Phys..

[cit26] Aguado A., Tablero C., Paniagua M. (1998). Comput. Phys. Commun..

[cit27] Varandas A. J. C., Poveda L. A. (2006). Theor. Chem. Acc..

[cit28] Martínez-Núñez E., Varandas A. J. C. (2001). J. Phys. Chem. A.

[cit29] Li Y. Q., Song Y. Z., Varandas A. J. C. (2015). Eur. Phys. J. D.

[cit30] Li Y. Q., Varandas A. J. C. (2010). J. Phys. Chem. A.

[cit31] Li Y. Q., Ma F. C., Sun M. T. (2013). J. Chem. Phys..

[cit32] Li Y. Q., Yuan J. C., Chen M. D., Ma F. C., Sun M. T. (2013). J. Comput. Chem..

[cit33] Li Y. Q., Varandas A. J. C. (2010). J. Phys. Chem. A.

[cit34] Li Y. Q., Song Y. Z., Song P., Li Y. Z., Ding Y., Sun M. T., Ma F. C. (2012). J. Chem. Phys..

[cit35] Biglari Z., Shayesteh A., Maghari A. (2014). Comput. Theor. Chem..

[cit36] Reddy R. R., Nazeer Ahammed Y., Rama Gopal K., Baba Basha D. (2004). J. Quant. Spectrosc. Radiat. Transfer.

[cit37] Kowalski K., Piecuch P. (2001). J. Chem. Phys..

[cit38] Hakalla R., Kepa R., Szajna W., Zachwieja M. (2006). Eur. Phys. J. D.

[cit39] Hechtfischer U., Williams C. J., Lange M., Linkemann J., Schwalm D., Wester R., Wolf A., Zajfman D. (2002). J. Chem. Phys..

[cit40] Varandas A. J. C. (1996). J. Chem. Phys..

[cit41] Song Y. Z., Zhang Y., Zhang L. L., Gao S. B., Meng Q. T. (2015). Chin. Phys. B.

[cit42] Yang C. L., Huang Y. J., Zhang X., Han K. L. (2003). J. Mol. Struct.: THEOCHEM.

[cit43] Huber K. P. G. H. , NIST Chemistry WebBook69, 2001, see http://webbook.nist.gov/chemistry

[cit44] Huber K. P. and Herzberg G., Molecular Spectra and Molecular Structure IV: Constants of Diatomic Molecules, Van Nostrand Reinhold, New York, 1979

[cit45] Balakrishnan A., Smith V., Stoicheff B. P. (1992). Phys. Rev. Lett..

[cit46] Badger R. M. (1935). J. Chem. Phys..

[cit47] Brinkmann N. R., Richardson N. A., Wesolowski S. S., Yamaguchi Y., Schaefer III H. F. (2002). Chem. Phys. Lett..

[cit48] Jensen P., Brumm M., Kraemer W. P., Bunker P. R. (1995). J. Mol. Spectrosc..

[cit49] Varandas A. J. C. (1987). Chem. Phys. Lett..

[cit50] Han K. L., He G. Z., Lou N. Q. (1996). J. Chem. Phys..

[cit51] Chen M. D., Han K. L., Lou N. Q. (2003). J. Chem. Phys..

[cit52] Skouteris D., Castillo J. F., Manolopoulos D. E. (2000). Comput. Phys. Commun..

[cit53] Pino I., Martinazzob R., Tantardini G. F. (2008). Phys. Chem. Chem. Phys..

[cit54] Karplus M., Porter R. N., Sharma R. D. (1965). J. Chem. Phys..

[cit55] Luca A. , Borodi G. and Gerlich D., Photonic, Electronic and Atomic Collisions, World Scientific, Singapore, 2006

[cit56] Federer W., Villinger H., Howorka F., Lindinger W., Tosi P., Bassi D., Ferguson E. (1984). Phys. Rev. Lett..

