# Supplementary material for: Accurate global potential energy surface for the ground state of CH2+ by extrapolation to the complete basis set limit
Source: RSC Adv. 2018 Apr 11;8(25):13635–42. doi: 10.1039/c8ra02228c (PMC9079815; doi:10.1039/c8ra02228c)
Supplement: RA-008-C8RA02228C-s001 [file RA-008-C8RA02228C-s001.pdf]

Electronic Supplementary Material (ESI) for RSC Advances.
This journal is © The Royal Society of Chemistry 2018

Accurate global potential energy surface for ground state of CH+ 2by extrapolation to the complete basis set limit

L. Guo,[1] H. Y. Ma,[1] L. L. Zhang,[2] Y. Z. Song[2†] and Y. Q. Li[1*]

[1]Department of Physics, Liaoning University, Shenyang 110036, China

[2]College of Physics and Electronics, Shandong Normal University, Jinan 250014, China

† E-mail address: yzsong@sdnu.edu.cn

* E-mail address: yqli@lnu.edu.cn

Table 1. Fitted parameters of two-body energy term in Eq. (8) and (9), the root-mean square derivations (ΔE_{RMSD}) are in kcal/mol.

	CH^+ ($X\ ^1\Sigma^+$)	H_2 ($X\ ^1\Sigma+$ g)
a_0	0.68749081×10^3	0.10524396×10^1
a_1	-0.39969300×10^1	-0.58820524×10^{-1}
a_2	0.57825124×10^2	-0.98926851×10^1
a_3	-0.84050850×10^3	0.76642439×10^2
a_4	0.95974671×10^4	0.39114853×10^3
a_5	-0.70346709×10^5	0.13715868×10^4
a_6	0.32377985×10^6	-0.32031792×10^4
a_7	-0.83941570×10^6	0.44814936×10^4
a_8	0.95845581×10^6	-0.28282256×10^4
β_1	9.1180	2.2730
β_2	1.4160	1.1280
ΔE_{RMSD}	0.049444	0.0046165

Table 2. Parameters and reference geometries in the three-body energy term of Eq. (10) for ground state CH_2^+ PES.

Coefficients	$P^{(1)}$	$P^{(2)}$	$P^{(3)}$
γ(j)1/a_0^{-1}	0.4	1.4	1.4
γ(j) 2/a_0^{-1}	1.0	0.5	0.5
γ(j) 3/a_0^{-1}	1.0	0.6	0.6
R(j) 1,ref/a_0	1.5	4.0	4.0
R(j) 2,ref/a_0	2.5	3.0	3.0
R(j) 3,ref/a_0	3.5	3.5	3.5

Table 3. Linear coefficients in the three-body energy term which used in Eq. (10).

Coefficients	$P^{(1)}$	$P^{(2)}$	$P^{(3)}$
C_1/a_0^0	3.0414614822	0.4875449048	−0.1459518090
C_2/a_0^{-1}	1.5884754507	1.6796021351	−2.3131550256
C_3/a_0^{-1}	0.7829609099	0.2054445499	0.2123848344
C_4/a_0^{-2}	−0.0246501521	−0.4914758788	0.1145048312
C_5/a_0^{-2}	−0.2024395298	0.1924919711	0.0408065987
C_6/a_0^{-2}	0.2108929703	−0.2683057446	−0.5846008578
C_7/a_0^{-2}	−0.7103998370	0.0602930567	0.2476510112
C_8/a_0^{-3}	0.3094103241	0.0244719271	−0.0268377039
C_9/a_0^{-3}	0.0109815694	−0.0844730123	−0.0063074906
C_{10}/a_0^{-3}	−0.0788051045	−0.1326368487	−0.0208125604
C_{11}/a_0^{-3}	0.0416220187	0.0441455708	−0.0138245702
C_{12}/a_0^{-3}	0.0068967088	0.0003386145	0.0035484502
C_{13}/a_0^{-3}	−0.0038626275	−0.0112047593	−0.0018380159
C_{14}/a_0^{-4}	0.0039340281	−0.0016742396	−0.0042785057
C_{15}/a_0^{-4}	−0.0075607514	0.0033283700	0.0003176933
C_{16}/a_0^{-4}	−0.0060301500	0.0005363062	−0.0043942503
C_{17}/a_0^{-4}	0.0052856716	0.0061124842	−14.1532406443
C_{18}/a_0^{-4}	5.0763090893	−1.4381877721	−3.0263766302
C_{19}/a_0^{-4}	−1.9182964917	−0.0399935171	3.0822769058
C_{20}/a_0^{-4}	0.4921191941	0.9046797875	−0.4342600437
C_{21}/a_0^{-4}	1.5730246304	−1.0249359646	−0.3629080478
C_{22}/a_0^{-4}	−0.1786355977	0.9235218936	0.0778153262
C_{23}/a_0^{-5}	0.2044899335	0.2890371718	−0.6998826527
C_{24}/a_0^{-5}	1.0547501187	−0.1902059608	0.1539080720
C_{25}/a_0^{-5}	−0.0226814080	0.0349281254	0.1023863743
C_{26}/a_0^{-5}	0.0645015955	−0.0570632072	−0.0822206224
C_{27}/a_0^{-5}	−0.0485249582	0.3144496160	−0.0586076591
C_{28}/a_0^{-5}	−0.1386100557	0.0057042405	0.0468758646
C_{29}/a_0^{-5}	−0.0028928535	−0.0037712097	0.0395711107
C_{30}/a_0^{-5}	0.0034986458	0.0038618764	0.0078831520
C_{31}/a_0^{-5}	−0.0010079582	−0.0084766494	0.0109183990
C_{32}/a_0^{-5}	0.0102753355	0.0183766933	−0.0139180265
C_{33}/a_0^{-5}	0.0514820122	−0.0035346667	−0.0120342665
C_{34}/a_0^{-5}	−0.0124267789	−1.8744679178	0.4400172916
C_{35}/a_0^{-6}	−3.4596202229	0.0087416310	−0.7549804994
C_{36}/a_0^{-6}	−1.0681863361	0.4955391941	0.0770823719

C_{37}/a_0^{-6}	−0. 5476732009	−0. 2020269535	−0. 4277248710
C_{38}/a_0^{-6}	0. 6432499961	−0. 9956212552	0. 0470613833
C_{39}/a_0^{-6}	−0. 0930917343	−0. 1228740323	−0. 0853562138
C_{40}/a_0^{-6}	0. 0404700067	0. 0455836525	−0. 3144261128
C_{41}/a_0^{-6}	−0. 0424467672	0. 0709904072	0. 0076602305
C_{42}/a_0^{-6}	0. 0016992660	−0. 0432866548	−0. 0135017811
C_{43}/a_0^{-6}	−0. 0180500284	−0. 0013874189	0. 0238691012
C_{44}/a_0^{-6}	−0. 0382553716	0. 0053250063	0. 0454531895
C_{45}/a_0^{-6}	−0. 0539613489	−0. 0019129031	0. 0015443974
C_{46}/a_0^{-6}	−0. 0046509249	−0. 0136196496	−0. 0017237226
C_{47}/a_0^{-6}	−0. 0040444248	−0. 0061137839	−0. 0002850657
C_{48}/a_0^{-6}	0. 0008934090	0. 0005727100	−0. 0107846696
C_{49}/a_0^{-6}	−0. 0065497196	0. 0029050872	−0. 0203324375
C_{50}/a_0^{-6}	0. 0061373270	−0. 0014896807	0. 0056284355
